# Role of ATF-2 in regulation of epithelial–mesenchymal transition and radio-sensitivity of A549 cells mediated by secreted soluble factors

**DOI:** 10.1093/jrr/rrt162

**Published:** 2014-03

**Authors:** Sejal Desai, S. Laskar, B. N. Pandey

**Affiliations:** 1 Radiation Biology and Health Sciences Division, Bhabha Atomic Research Centre, Mumbai 400085, India; 2 Department of Radiation Oncology, Tata Memorial Hospital, Dr Ernest Borges Marg, Parel, Mumbai 400012, India

**Keywords:** Epithelial–mesenchymal transition, gamma radiation, radio-resistance, tumor secreted soluble factors, ATF-2

## Abstract

**Introduction:**

Epithelial–mesenchymal transition (EMT) is known to be a crucial event whereby cancer cells acquire the traits needed to execute multiple steps of metastasis [ 1]. Intriguingly, epithelial cancer cells undergoing EMT also exhibit increased radioresistance [ 2]. EMT is a dynamic process and is triggered by various micro-environmental stimuli, out of which secreted soluble factors play a major role [ 3]. Herein, we have investigated the effect of self-conditioned medium (CM) on the human lung carcinoma A549 cells and the underlying mechanism.

**Material and methods:**

A549 cells were obtained from National Centre for Cell Sciences, Pune, and cultured in DMEM with 10% FCS and antibiotics at 37°C in 5% CO_2_/air. To get CM, 1 × 10^6^ A549 cells were grown in complete medium. After 48 h of incubation, media were harvested referred as CM. Media alone without cells were incubated under the same conditions served as control termed as plain medium (PM) [ 4]. A549 cells grown in PM/CM subjected to immunofluorescence staining for E-cadherin, vimentin and actin. Motility scratch assay and matrigel invasion assay were performed to study the invasiveness of A549 cells in PM/CM. In some cases, cells were pretreated with inhibitors against MAPKs. To pinpoint the soluble factor(s) in CM responsible for initiating the invasiveness in A549 cells, CM was pretreated with neutralizing antibodies against IL-8, VEGF and IL-1β.

**Results and discussion:**

We found that CM induced a series of changes associated with EMT in A549 cells such as change in shape from cuboidal to elongated spindle shape, decrease in E-cadherin, increase in vimentin and enhanced actin polymerization with decreased cell–cell contact. CM also promoted motility, invasion and anchorage-independent growth in A549 cells. Neutralizing IL-8, IL-1β and VEGF in CM significantly inhibited motility and invasion. Moreover, JNK and p38 activated by CM in A549 cells played important role in invasiveness. In downstream signalling, CM activated ATF-2 by phosphorylating it at Thr69 and 71. Knockdown of ATF-2 using siRNA abrogated the CM-enhanced motility and invasion. It has been reported that EMT not only accelerates cancer metastasis but may also result in resistance against therapeutic agents [ 2, 5]. We therefore investigated the effect of CM on radioresistance of A549 cells. Our results showed that A549 cells grown in CM followed by gamma irradiation exhibited higher clonogenic survival and lower DNA damage measured in terms of gamma-H2AX foci. Interestingly, these CM-treated A549 cells in response to radiation showed phosphorylation of ATF-2 at Ser 490/498 (not at Thr69/71), which was co-localized with gamma-H2AX foci. These studies suggest the dual role of ATF-2, i.e. as EMT potentiating transcription factor and DNA damage response protein in lung cancer cells stimulated with soluble factors, which can be exploited as potential therapeutic target for cancer radiotherapy.
